# The role of G*α*_O_‐mediated signaling in the rostral ventrolateral medulla oblongata in cardiovascular reflexes and control of cardiac ventricular excitability

**DOI:** 10.14814/phy2.12860

**Published:** 2016-08-15

**Authors:** Richard Ang, Joel Abramowitz, Lutz Birnbaumer, Alexander V. Gourine, Andrew Tinker

**Affiliations:** ^1^ William Harvey Heart CentreBarts & The London School of Medicine and DentistryLondonUK; ^2^ Department of Neuroscience, Physiology and PharmacologyUniversity College LondonLondonUK; ^3^ Division of Intramural ResearchNational Institute of Environmental Health SciencesResearch Triangle ParkNorth Carolina

**Keywords:** Autonomic nervous system, blood pressure, cardiac excitability, G proteins, rostral ventral lateral medulla

## Abstract

The heart is controlled by the sympathetic and parasympathetic limbs of the autonomic nervous system with inhibitory signaling mechanisms recruited in both limbs. The aim of this study was to determine the role of inhibitory heterotrimeric G proteins in the central nervous mechanisms underlying autonomic control of the heart and its potential role in arrhythmogenesis. Mice with conditional deletion of the inhibitory heterotrimeric G protein G*α*_O_ in the presympathetic area of the rostral ventral lateral medulla (RVLM) were generated to determine the role of GαO‐mediated signalling in autonomic control and electrophysiological properties of the heart. G*α*_O_ deletion within the RVLM was not associated with changes in heart rate (HR) or the arterial blood pressure at rest (home cage, normal behavior). However, exposure to stressful conditions (novel environment, hypoxia, or hypercapnia) in these mice was associated with abnormal HR responses and an increased baroreflex gain when assessed under urethane anesthesia. This was associated with shortening of the ventricular effective refractory period. This phenotype was reversed by systemic beta‐adrenoceptor blockade, suggesting that G*α*_O_ depletion in the RVLM increases central sympathetic drive. The data obtained support the hypothesis that G*α*_O_‐mediated signaling within the presympathetic circuits of the RVLM contributes to the autonomic control of the heart. G*α*_O_ deficiency in the RVLM has a significant impact on cardiovascular responses to stress, cardiovascular reflexes and electrical properties of the heart.

## Introduction

The heart is controlled by the autonomic nervous system via its two functional limbs: the sympathetic and parasympathetic (Spyer 1994). Sympathovagal imbalance can trigger ventricular arrhythmias in patients with inherited “channelopathies” such as the long QT syndrome, Brugada syndrome, catecholaminergic polymorphic ventricular tachycardia (Verrier and Antzelevitch 2004), and also in patients with ischemic heart disease (Nolan et al. 1998). Sympathetic denervation has been used as an adjunct to pharmacotherapy with beta‐adrenoceptor antagonists in the management of patients with resistant ventricular arrhythmias (Vaseghi et al. 2014). However, the efficacy of current therapeutic strategies remains limited, perhaps reflecting the fact that only the downstream peripheral mechanisms are targeted.

Heterotrimeric G proteins mediate signaling via G‐protein coupled receptors (GPCRs). GPCRs are involved in autonomic control at the level of the heart via beta adrenergic and muscarinic receptors signaling via stimulatory G*α*_S_ and inhibitory G*α*_i/o_ proteins. There appears to be specificity of G*α* protein subtypes in vivo. Zuberi et al. (2008) have demonstrated the importance of the inhibitory G proteins G*α*_i2_ and G*α*_O_ in the autonomic control of the heart using global knockout (KO) mouse models. We have recently shown that G*α*_i2_ is important in mediating parasympathetic influences at the level of the cardiac conduction tissue, probably by coupling to M2 muscarinic receptors (Sebastian et al. 2013). What remains unclear is the exact role played by the inhibitory G proteins in the central nervous mechanisms of autonomic regulation of the heart.

G*α*_O_ protein is a key inhibitory G protein subtype in the central nervous system (CNS) (Sternweis and Robishaw 1984) including the brainstem (Parker et al. 2012). Mice with global deletion of G*α*_O_ are tachycardic and display loss of diurnal rhythm in heart rate (HR) and selective loss of the low‐frequency component of HR variability with preserved total power (Zuberi et al. 2008). However, carbachol still had a negative chronotropic effect in this model, suggestive of increased sympathetic activity (Zuberi et al. 2008). The rostral ventrolateral medulla (RVLM) contains principal presympathetic circuits which generate central sympathetic drive (Head and McCarty 2002; Nalivaiko et al. 2005). Increased activity of the RVLM neurons has been implicated in the development and progression of cardiovascular diseases associated with enhanced central sympathetic drive including hypertension (Marina et al. 2015) and heart failure (Marina et al. 2011). We hypothesized that the inhibitory influences mediated via G*α*_O_ proteins in the RVLM are important in controlling (restricting) sympathetic tone. This would broadly impact on the blood pressure homeostasis and cardiac function, particularly HR and ventricular excitability. To test this hypothesis, we generated mice with conditional deletion of G*α*_O_ in the RVLM and studied their cardiovascular phenotype, cardiovascular reflexes, and ventricular excitability in vivo.

## Methods

### Murine husbandry

Mice were maintained in an animal core facility under the UK Home Office guidelines relating to animal welfare. All procedures were approved by the local animal care and use committee and performed in accord with the UK Home Office regulations. All mice were kept in a temperature‐controlled environment (21–24°C) with 12/12 h light/dark cycle. Animals were allowed ad libitum access to standard rodent chow and drinking water. Mice were studied between 8 and 12 weeks of age.

### Experimental animals

G*α*_O_ flx/flx mice on a Sv129 background with coding exons 5–6 of the G*α*_O_ allele on chromosome 8 flanked by loxP sites were generated using homologous recombination in mouse ES cells (Ustyugova et al. 2012). Gene targeting strategies, genotyping primers, and confirmation of selective deletion of G*α*_O_ at the protein level from various tissues including the brain using western blot approaches have been previously described (Ustyugova et al. 2012). Mice with conditional deletion of G*α*_O_ in the RVLM were then generated following microinjections of adenoviral vectors (AVV) coexpressing Cre and GFP under the control of a CMV promoter (Cre/GFP‐AVV) into the RVLM of G*α*_O_ flx/flx mice. Control mice were produced using GFP‐only‐expressing AVV under the control of the same CMV promoter (GFP‐AVV). These vectors have been described previously (LaVallie et al. 2006; Lopez et al. 2008) and were obtained from Viraquest (USA). In preliminary studies, quantitative RT‐PCR using TaqMan gene expression assays was performed at sites of injections to confirm knockdown of G*α*_O_ expression and determine the optimum dilution of viral titers to be used. Immunohistochemistry was performed at the end of all the experiments to determine colocalization of GFP expression with tyrosine hydroxylase (TH) immunoreactivity in the RVLM.

### Experiments in conscious freely moving mice

**Figure 1:**
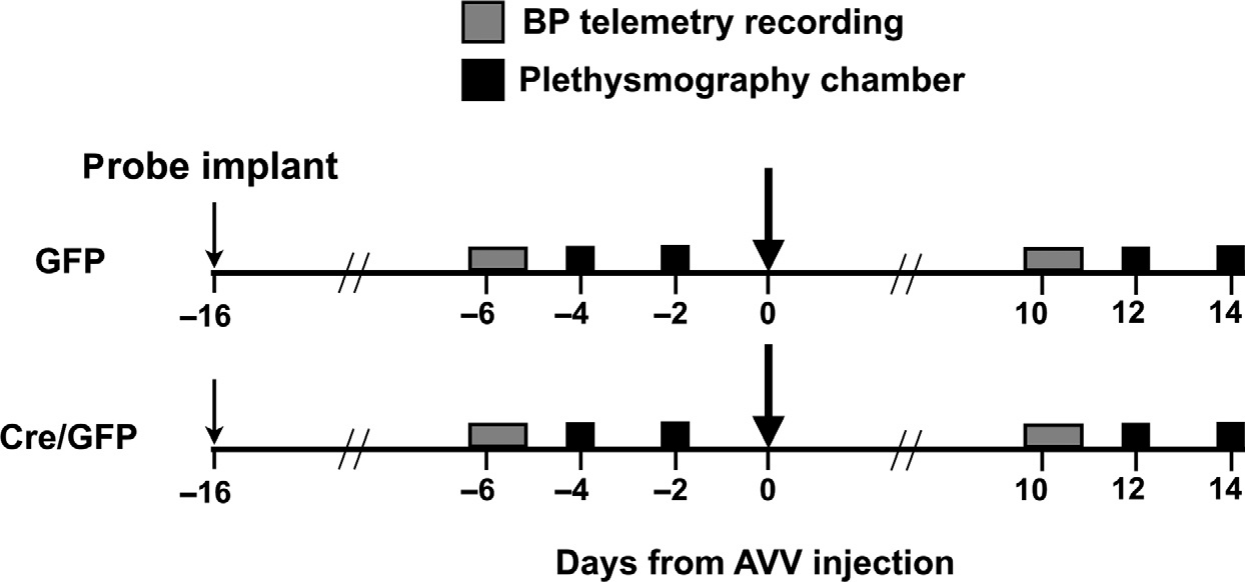
Experimental protocol for conscious experiments. Figure showing timeline of conscious experimental protocols. Adenoviral vectors (AVV) injection to the brainstem is performed at Day 0.

Conscious freely behaving G*α*_O_ flx/flx mice were first studied in their normal housing conditions and in a plethysmography chamber. Figure 1 illustrates the experimental protocol used.

### Biotelemetry recordings of the systemic arterial blood pressure

Blood pressure (BP) telemetry probe implantation and recording techniques have been described in detail previously (Aziz et al. 2014). Briefly, the left carotid artery was cannulated with a gel‐filled catheter of the BP telemetry probe (model PA‐C10; Data Sciences International, St Paul, MN) which allows beat‐to‐beat biotelemetry recording of the arterial BP in conscious freely moving mouse. Heart rate (HR) was derived from the BP recording trace.

### Recording of the 24‐h BP and HR profile

Animals were allowed to recover for at least 10 days after the transmitter implantation. Experiments were performed in a temperature‐controlled environment (21–24°C) with 12 h/12 h light/dark cycle. The animals were allowed to acclimatize to the experimental conditions during the recovery period from surgery. For serial BP and HR measurements, 15‐second‐long samples were taken every 30 min over 24 h using a “scheduled sampling” protocol. From the recorded dataset, it was possible to determine the diurnal changes in BP and HR during the day and night and also calculate the mean values.

### Whole body plethysmography

Mice with telemetry probes implanted were also exposed to hypoxic and hypercapnic conditions. Respiratory rate (*f*_R_, breaths min^−1^), tidal volume (*V*_T_, arbitrary unit g^−1^), and minute ventilation (*V*_*E*_) ((*f*_R_ × *V*_T_); arbitrary unit min^−1^ g^−1^) were measured using whole body plethysmography as described in detail previously (Onodera et al. 1997; Rong et al. 2003; Trapp et al. 2011) and BP and derived HR simultaneously recorded using radiotelemetry. Briefly, the mouse was placed in a plexiglass recording chamber (~200 mL) that is in turn placed on a telemetry recording mat. The chamber was flushed continuously with a mixture of 79% nitrogen and 21% oxygen (unless otherwise required by protocol) at a rate of ~1 L min^−1^. Concentrations of O_2_ and CO_2_ in the chamber were monitored online using a fast‐response O_2_/CO_2_ monitor (Morgan Scientific, Haverhill, MA).

The animals were allowed at least 20 min to acclimatize to the chamber environment at normoxia/normocapnia (21% O_2_, 79% N_2_, and <0.3% CO_2_) before measurements of baseline ventilation were taken. Hypoxia was induced by lowering the O_2_ concentration in the inspired air down to 10% for 5 min. After 5 min, the O_2_ concentration was then brought back up to 21% for a further 5 min. In a separate experiment, normoxic hypercapnia was induced by titrating CO_2_ into the respiratory mixture up to a level of 3% or 6% (lowering N_2_ accordingly) for 5 min at each CO_2_ level. The measurements were taken during the last 2 min before exposure to the stimulus and during the 2‐min period at the end of each stimulus, when breathing has stabilized. Simultaneous continuous recordings of changes in BP, HR, *f*_R_ and *V*_T_ during hypoxic and hypercapnic challenges were obtained.

### In vivo experiments under general anesthesia

A separate cohort of G*α*_O_ flx/flx mice were studied in the anesthetized state after Cre/GFP AVV or control GFP AVV injections into the RVLM. Mice were anesthetized with urethane (1.3 g kg^−1^ ip). The depth of anesthesia was monitored using the stability of BP, HR, and lack of flexor responses to a paw pinch and supplemental anesthesia was given as required. Body temperature was maintained at 37.0 ± 0.2°C using a servo‐controlled heating pad. Tracheostomy was performed to facilitate ventilation in spontaneously breathing mice. Needle electrodes were placed in a lead II configuration to record surface ECG (sampled at 2 kHz, amplified × 50–100, and filtered to a bandwidth between 5 and 100 Hz with 50 Hz notch filtering).

### Determining baroreflex sensitivity

The right and left jugular veins were cannulated with polyethylene tubing (PE‐10) for administration of phenylephrine (0.5–2.5 mg kg^−1^ iv in 2–10 *μ*L saline) or sodium nitroprusside (SNP, 0.1–1 mg kg^−1^ iv in 1–10 *μ*L saline), respectively. The left carotid artery was cannulated with saline‐filled polyethylene tubing (mechanically stretched PE‐10 connected to PE‐50) connected to a pressure transducer to measure arterial BP (sampled at 2 kHz). Changes in the systemic arterial BP were induced by alternately administrating phenylephrine and SNP and varying the volumes of injectate. The baroreflex sensitivity curve was then derived as described by Head and McCarty (2002). The peak changes in arterial BP and HR were determined following the administration of each drug and the changes from basal values were then calculated for each pair of data points and scaled back to absolute BP and HR by the average of all basal values recorded. About 10–15 data points were obtained across the range of BP‐HR for each animal and the data points were then fitted to the following logistic equation using a least squares iterative routine on MatLab (R2010b, MathWorks, Natick, MA).

$$\text{HR}=P1+P2/[1+e^{P3(\mathrm{BP}-P4)}]$$

where P1 = lower HR plateau, P2 = HR range, P3 = a curvature coefficient which is independent of range, and P4 = BP_50_, that is, the BP at half the HR range. The average baroreflex gain (BRG) or slope of the curve between the two inflection points is given by BRG = −P2 × P3/4.56 and the upper plateau = P1 + P2.

### Ventricular electrophysiology

A separate group of mice underwent programmed electrical assessment of ventricular excitability. An octapolar 1.1F cardiac electrophysiology catheter was inserted into the right ventricular apex through the right internal jugular vein to pace the heart as previously described (Zuberi et al. 2010; Machhada et al. 2015). Cardiac pacing using extrastimulation protocols was performed with an S88‐Grass stimulator to record the ventricular effective refractory period at a basic drive train of 750 beats per minute (VERP_750_).

### Statistical analysis

Data are reported as means ± standard error of the mean (SEM) unless stated otherwise. For determination of statistical significance between groups, two‐tailed Student's *t* test was used for parametric data with a normal distribution and the Mann–Whitney *U* Test was used as a nonparametric test. Fisher's exact test was used for comparing categorical data. For data with repeated sampling between multiple groups, mixed‐level modeling using both fixed and random effects was used to compare the mean effect of each group. Analysis of variance with Bonferroni multiple comparison test was used to compare the difference of effect between groups. In all the instances, *P *<* *0.05 was considered significant.

## Results

### Generation of mice with conditional deletion of Gα_O_ deletion in the RVLM and controls

**Figure 2:**
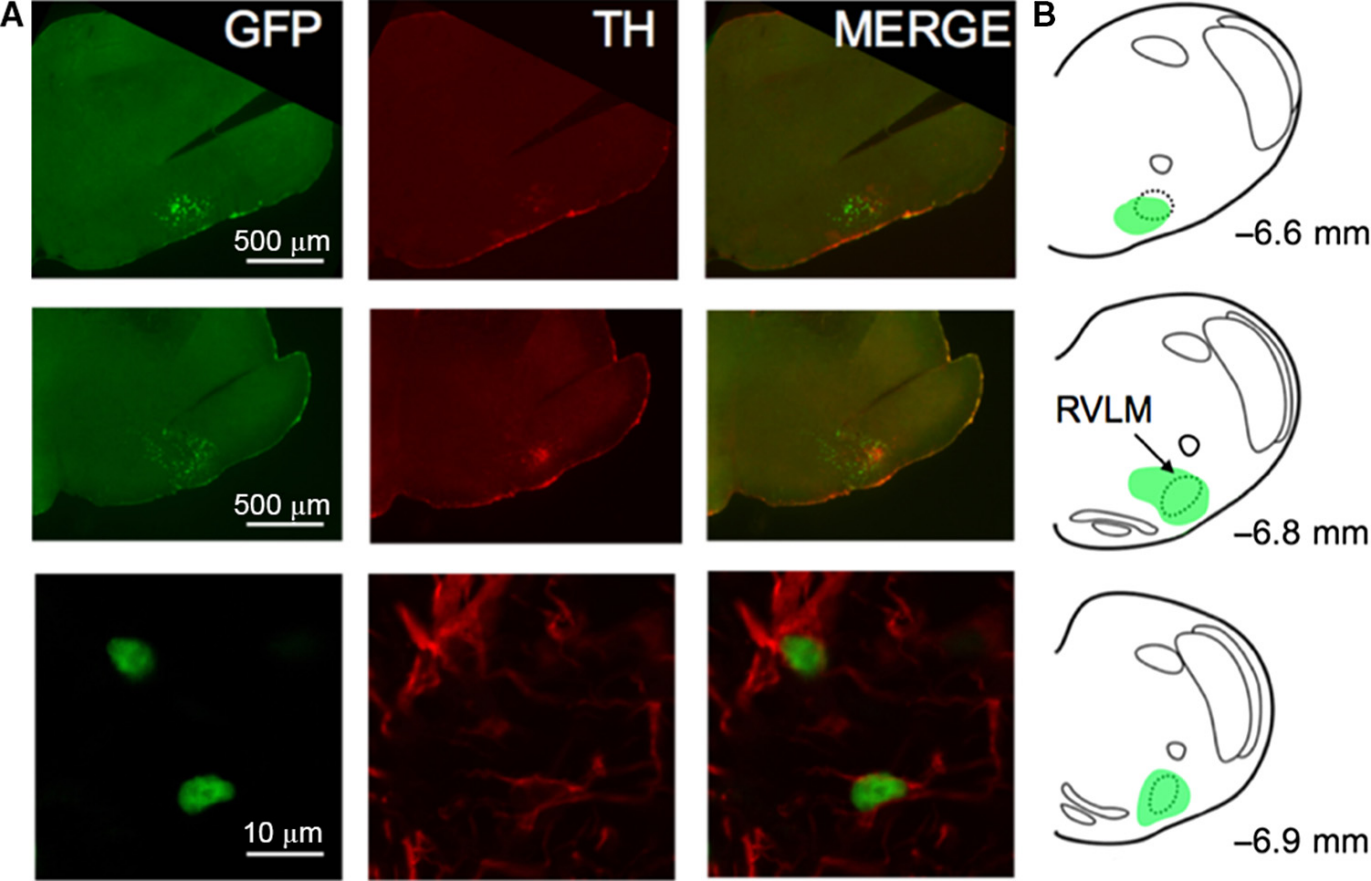
Green fluorescent protein (GFP) expression in TH‐positive neurons in the brainstem. (A), GFP expression in the C1 region of the rostral ventral lateral medulla (RVLM) (TH, tyrosine hydroxylase). Bottom image is a high magnification micrograph showing two TH‐positive neurons expressing GFP. (B), Schematic drawing of the mouse brainstem in a series of coronal projections illustrating the representative extent of GFP expression in relation to the anatomic location of the RVLM presympathetic circuits. Numbers indicate distance from Bregma. GFP expression was highest in the ventrolateral medullary regions located at −6.80 mm from Bregma.

Using 1:100 dilutions of Cre/GFP AVV (6 × 10^10^ PFU) and GFP AVV (3 × 10^10^ PFU), G*α*_O_ expression at the sites of injections was reduced by 79% in mice post‐Cre/GFP‐AVV injections (relative expression 0.21, 95% CI 0.18–0.25) compared to 21% post‐GFP‐AVV injections (relative expression 0.79, 95% CI 0.75–0.82), when normalized to the expression in mice preinjections (*n* = 3 mice in triplicate for both groups). Figure 2 shows the representative brainstem distribution of GFP‐expressing cells relative to TH immunoreactivity.

### Diurnal profile of HR and BP changes in conditions of Gα_O_ deletion in the RVLM

**Figure 3:**
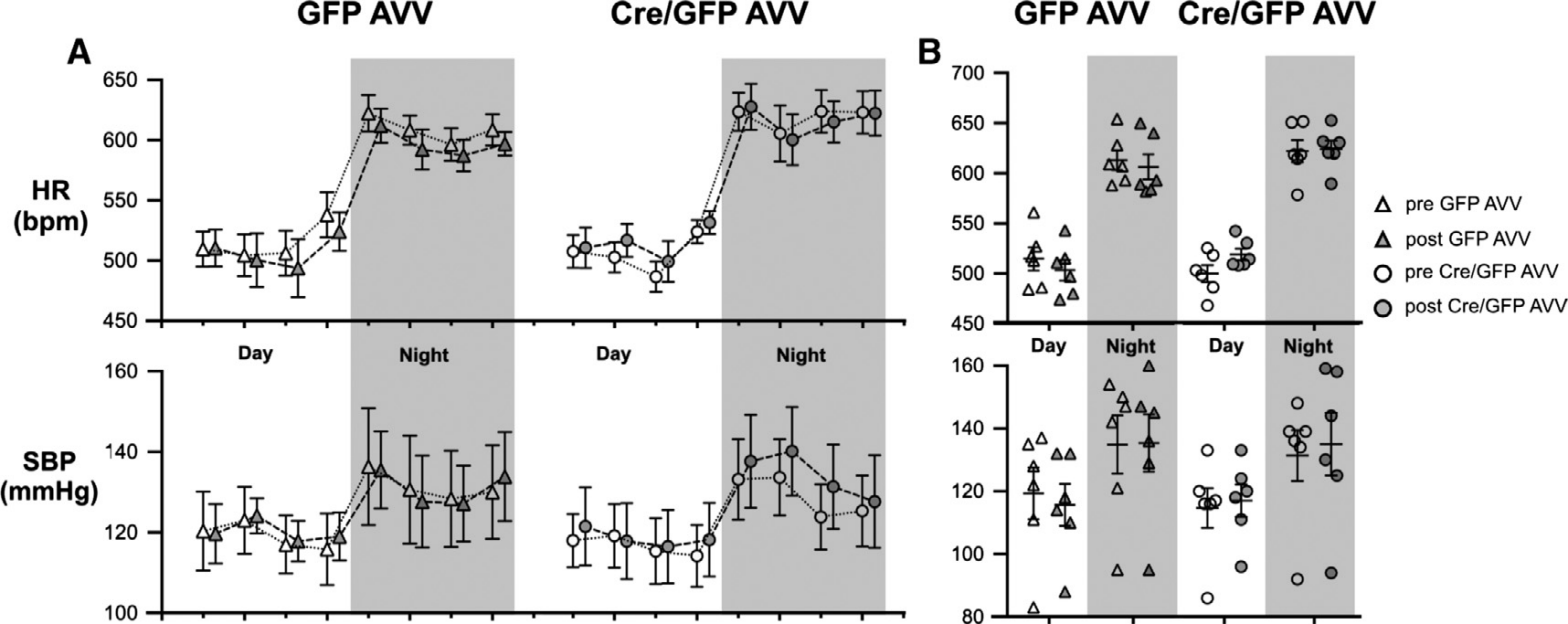
A 24‐h hemodynamic profile in conscious freely moving mice under normal housing conditions. (A) Heart rate (HR) and systolic blood pressure (SBP) of G*α*_O_ flx/flx mice pre‐ and post‐GFP and Cre/GFP adenoviral vectors (AVV) injections into the brainstem measured using biotelemetry over a 24‐h period. (B) Summary data of mean systolic arterial BP recorded (top) and HR (bottom) in G*α*_O_ flx/flx mice pre‐ and post‐GFP AVV (GFP) and Cre/GFP AVV (Cre) injections into the brainstem. Data expressed as mean ± SEM,* n* = 6 in all groups.

We first examined the 24‐h HR and BP profile of G*α*_O_ flx/flx mice pre‐ and post‐Cre/GFP AVV (*n* = 6) and control GFP AVV (*n* = 6) injections under normal housing conditions. There was a clear circadian variation in HR and systolic BP in G*α*_O_ flx/flx mice which was unchanged following injections of Cre/GFP AVV and control GFP AVV (Fig. 3).

### Hemodynamic and respiratory responses to hypoxia and hypercapnia in conditions of Gα_O_ deletion in the RVLM

**Figure 4:**
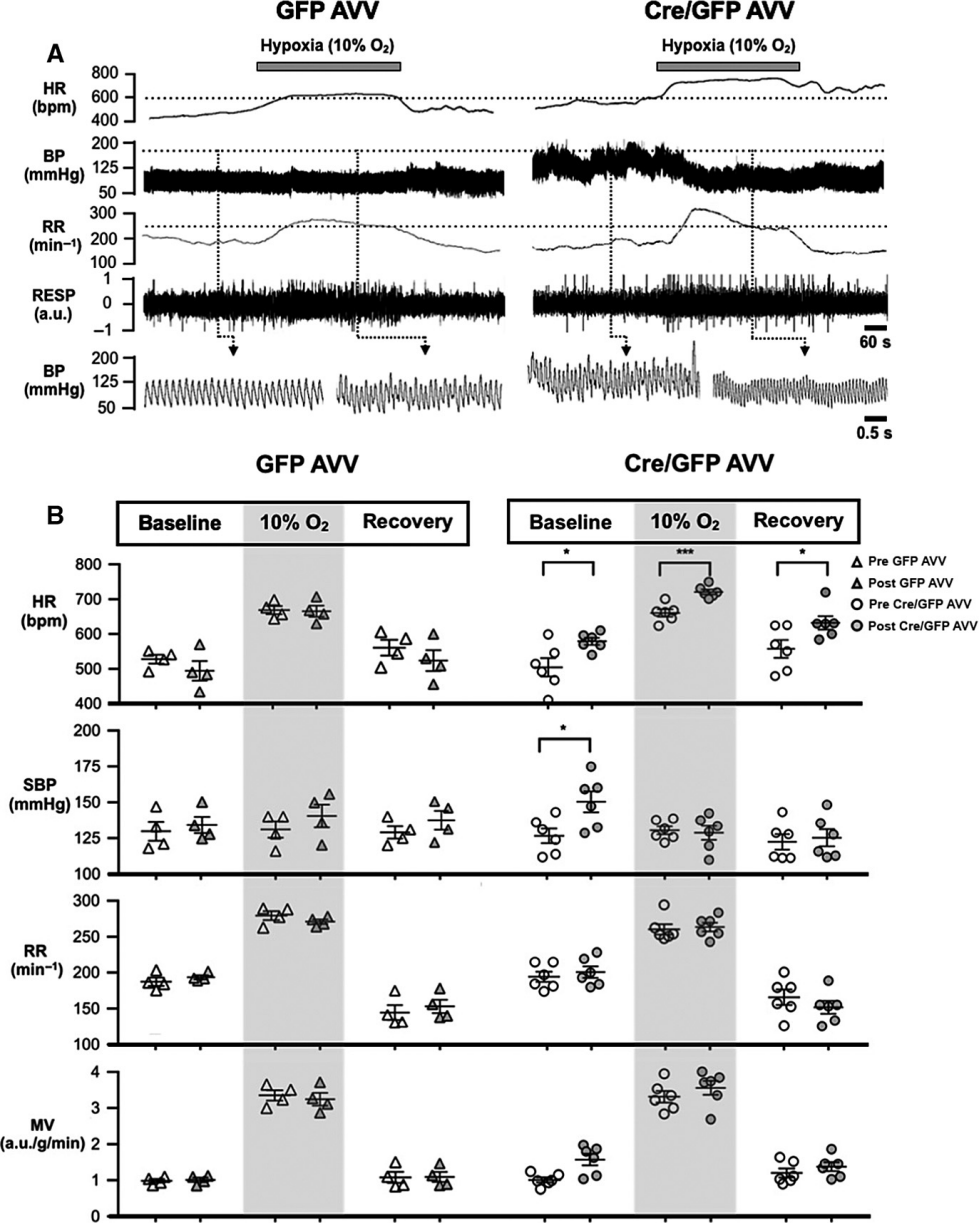
Hemodynamic and respiratory responses to hypoxia. (A) Representative heart rate (HR), arterial blood pressure (BP), respiratory rate (RR), and respiratory pressure (RESP) tracings of a G*α*_O_ flx/flx mouse post‐GFP adenoviral vectors (AVV) (left panel) and Cre/GFP AVV (right panel) injections, respectively, subjected to normoxia, hypoxia (10% inspired O_2_), and a recovery period. (B) HR, SBP, RR, and minute volume ventilation (MV) pre and post control GFP AVV (*n* = 4, left panel) and Cre/GFP AVV (*n* = 6, right panel) injections. Individual data and mean ± SEM are shown. *P <* *0.05, ***P <* *0.001. a.u., arbitrary units.

G*α*_O_ flx/flx mice were then studied in the plethysmography chamber. G*α*_O_ flx/flx mice responded to hypoxia (10% O_2_) with an increase in HR, respiratory rate, tidal volume, and minute ventilation, but there was no significant change in the systolic BP. There was posthypoxic depression of respiratory activity with reduced respiratory rate below baseline and normalization of HR and tidal volume. In conditions of G*α*_O_ deletion in the RVLM (post‐Cre/GFP AVV injections in G*α*_O_ flx/flx mice), the animals were more tachycardic at baseline (579 ± 10 vs. 504 ± 26 bpm, *P* = 0.023, mixed‐level modeling), during the hypoxic challenge (721 ± 7 vs. 656 ± 10 bpm, *P *<* *0.001) and during reoxygenation (633 ± 14 vs. 560 ± 26 bpm, *P* = 0.049) compared to the preinjection state. There was no difference in the change in HR during hypoxic challenge or recovery comparing pre‐ and post‐Cre/GFP AVV injections. G*α*_O_ flx/flx mice were also more hypertensive at baseline after the injections of Cre/GFP (152 ± 7 vs. 128 ± 5 mmHg, *P* = 0.025). There was a significant decrease in systolic BP on exposure to hypoxia (ΔSBP −22 mmHg, 95% CI −42 to −2 mmHg, *P* = 0.034, Bonferroni) which was maintained during recovery post‐Cre/GFP AVV injection. As a result, systolic BP during (132 ± 3 vs. 129 ± 5 mmHg, *P* = 0.671) and after hypoxia (124 ± 6 vs. 126 ± 6 mmHg, *P* = 0.796) were similar in pre‐ and post‐Cre/GFP AVV injections. Hypoxic ventilatory response was unaffected in conditions of G*α*_O_ deletion in the RVLM (Figs. 4 and 6A, Table 1).

**Table 1:** Summary data of hypoxia‐induced changes in hemodynamic and respiratory variables recorded in control (*n* = 4) and G*α*_O_‐floxed brainstem Cre mice (*n* = 6) pre‐ and postviral injections

	Baseline		10% O_2_		Recovery	
	GFP (*n* = 4)	Cre (*n* = 6)	GFP (*n* = 4)	Cre (*n* = 6)	GFP (*n* = 4)	Cre (*n* = 6)
Heart rate (BPM)						
Pre	527 ± 13	504 ± 26	665 ± 11	656 ± 10	559 ± 20	560 ± 26
Post	495 ± 28	**579 ± 10** *	665 ± 16	**721 ± 7** ***	524 ± 30	**633 ± 14** *
Systolic blood pressure (mmHg)						
Pre	129 ± 6	128 ± 5	124 ± 3	132 ± 3	129 ± 4	124 ± 6
Post	135 ± 5	**152 ± 7** *	126 ± 6	129 ± 5	128 ± 7	126 ± 6
Diastolic blood pressure (mmHg)						
Pre	62 ± 7	68 ± 6	62 ± 5	69 ± 5	62 ± 6	66 ± 6
Post	68 ± 6	**81 ± 8** *	65 ± 6	64 ± 4	63 ± 5	61 ± 7
Respiratory rate (min^−1^)						
Pre	185 ± 5	199 ± 7	280 ± 6	263 ± 7	145 ± 11	167 ± 10
Post	191 ± 3	199 ± 8	271 ± 3	266 ± 6	153 ± 9	155 ± 9
Minute volume (au g^−1^ min^−1^)						
Pre	1.0 ± 0.1	1.0 ± 0.1	3.4 ± 0.1	3.3 ± 0.2	1.1 ± 0.1	1.2 ± 0.1
Post	1.0 ± 0.1	1.1 ± 0.1	3.2 ± 0.2	3.5 ± 0.2	1.1 ± 0.1	1.4 ± 0.1

Data are expressed as means ± SEM. Values highlighted in bold are significantly different when compared to preinjection values. *P < 0.05, ***P < 0.001.

**Figure 5:**
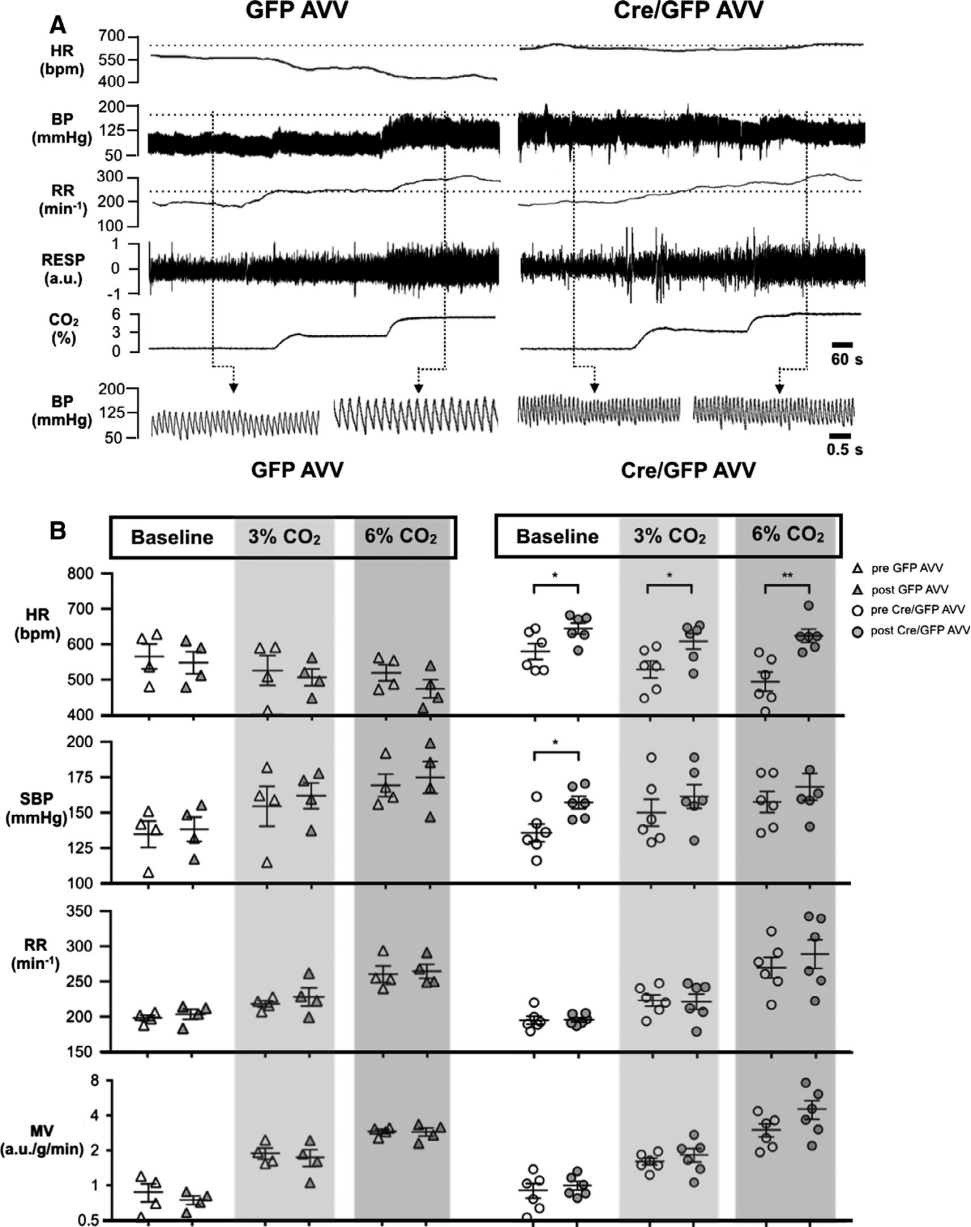
Hemodynamic and respiratory responses to hypercapnia. (A) Representative heart rate (HR) and arterial blood pressure (BP), respiratory rate (RR) and respiratory pressure (RESP) tracings of a G*α*_O_ flx/flx mouse post‐GFP adenoviral vectors (AVV) (left panel) and Cre/GFP AVV (right panel) injections, respectively, subjected to normoxia, 3% inspired CO_2_ and 6% inspired CO_2_. (B) HR, SBP, RR, and minute volume ventilation (MV) pre and post control GFP AVV (*n* = 4, left panel) and Cre/GFP AVV (*n* = 6, right panel) injections. Individual data and mean ± SEM are shown. *P <* *0.05, **P <* *0.01. abu, arbitrary units.

**Figure 6:**
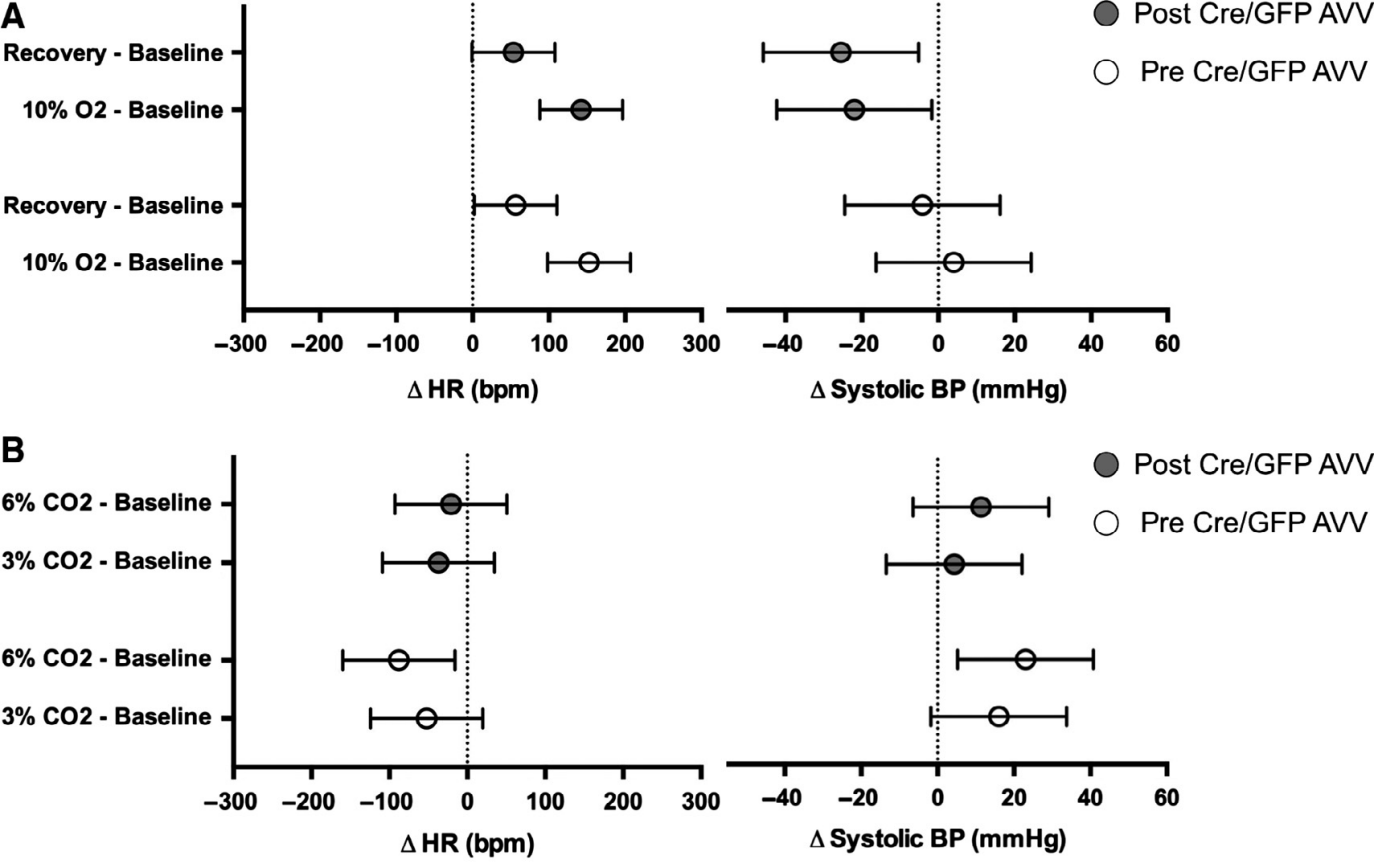
Changes in heart rate (HR) and systolic blood pressure (BP) in response to hypoxia and hypercapnia. Plots of the mean difference and 95% CI of HR and systolic BP (SBP) in G*α*_O_ flx/flx mice in response to (A) hypoxia and (B) hypercapnia.

G*α*_O_ flx/flx mice responded to 6% CO_2_ with a significant decrease in the HR (ΔHR‐88 bpm, 95% CI −160 to −16 bpm, *P* = 0.018), accompanied by an increase in the arterial BP (ΔSBP 23 mmHg, 95% CI 5–41 mmHg, *P* = 0.013). Hypercapnia also evoked stereotypic increases in minute ventilation. G*α*_O_ flx/flx mice displayed higher HR and systolic BP at baseline after acclimatization to the chamber environment post‐Cre/GFP AVV injections (654 ± 16 vs. 587 ± 23 bpm, *P* = 0.012; 159 ± 4 vs. 136 ± 6 mmHg, *P* = 0.012). HR depression during hypercapnia was attenuated post‐Cre/GFP AVV injections (ΔHR −21 bpm, 95% CI −93 to 51 bpm, *P* = 0.919), resulting in a consistently elevated HR throughout (3% CO_2_: 616 ± 23 vs. 539 ± 28 bpm, *P* = 0.049; 6% CO_2_: 633 ± 19 vs. 499 ± 27 bpm, *P* = 0.003). The elevation of systolic BP on exposure to 6% CO_2_ was also attenuated post‐Cre/GFP AVV injections (ΔSBP 11 95% CI −7 to 29 mmHg, *P* = 0.248) resulting in a higher systolic BP compared to preinjection (170 ± 10 vs. 159 ± 4 mmHg, *P* = 0.669). In summary, there is a significant difference in HR and systolic BP of mice at 6% CO_2_ compared to baseline preinjection of Cre/GFP AVV which is lost with G*α*_O_ deletion postinjection (Figs. 5 and 6B, Table 2).

**Table 2:** Summary data of hypercapnia‐induced changes in hemodynamic and respiratory variables recorded in control (*n* = 4) and G*α*_O_‐floxed brainstem Cre mice (*n* = 6) pre‐ and postviral injections

	Baseline		3% CO_2_		6% CO_2_	
	GFP (*n* = 4)	Cre (*n* = 6)	GFP (*n* = 4)	Cre (*n* = 6)	GFP (*n* = 4)	Cre (*n* = 6)
Heart rate (BPM)						
Pre	521 ± 41	587 ± 23	518 ± 22	539 ± 28	518 ± 22	499 ± 27
Post	507 ± 24	**654 ± 16** *	474 ± 26	**616 ± 23** *	474 ± 26	**633 ± 19** **
Systolic blood pressure (mmHg)						
Pre	135 ± 9	136 ± 6	155 ± 14	152 ± 9	169 ± 8	164 ± 9
Post	137 ± 8	**159 ± 4** *	160 ± 9	163 ± 9	173 ± 10	170 ± 10
Diastolic blood pressure (mmHg)						
Pre	65 ± 8	67 ± 7	65 ± 10	69 ± 8	66 ± 7	63 ± 8
Post	64 ± 7	**83 ± 6** *	63 ± 8	66 ± 9	64 ± 8	62 ± 9
Respiratory rate (min^−1^)						
Pre	198 ± 4	196 ± 6	218 ± 4	223 ± 8	260 ± 12	269 ± 14
Post	202 ± 7	201 ± 5	229 ± 12	221 ± 11	265 ± 10	307 ± 23
Minute volume (au g^−1^ min^−1^)						
Pre	1.0 ± 0.1	0.9 ± 0.1	1.9 ± 0.2	1.7 ± 0.1	2.9 ± 0.1	3.0 ± 0.4
Post	0.8 ± 0.2	1.0 ± 0.1	1.7 ± 0.3	1.9 ± 0.3	2.9 ± 0.3	4.7 ± 0.9

Data are expressed as means ± SEM. Values highlighted in bold are significantly different when compared to preinjection values. *P < 0.05, **P < 0.01.

There were no differences in hemodynamic and respiratory parameters in G*α*_O_ flx/flx mice post‐GFP AVV injections at baseline and during hypoxic and hypercapnic challenges (Figs. 4 and 5).

### Hemodynamic profile and baroreflex sensitivity in conditions of Gα_O_ deletion in the RVLM

**Figure 7:**
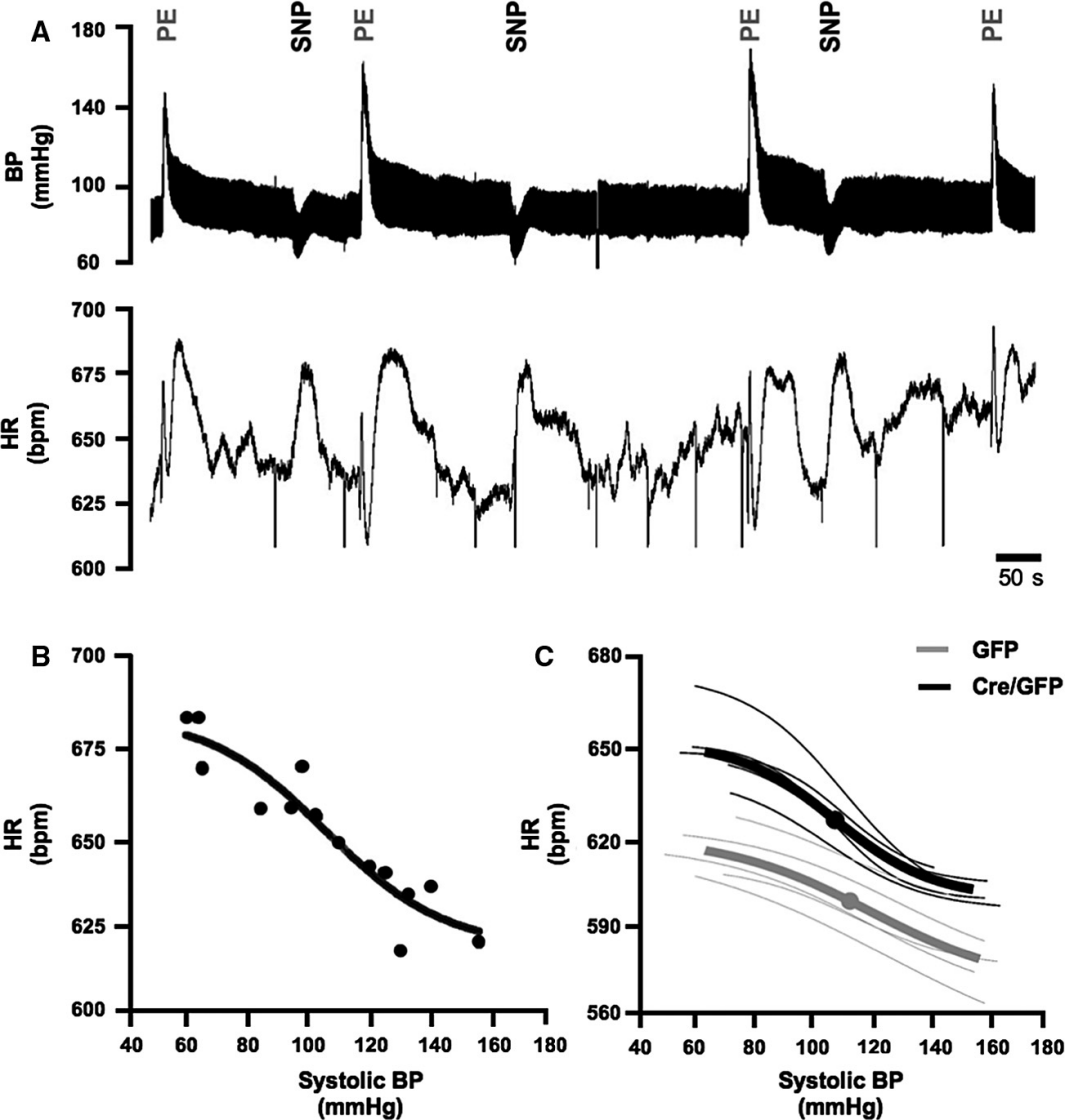
Baroreflex response under urethane anesthesia. (A) Representative tracings of arterial blood pressure (BP) and heart rate (HR) of a G*α*_O_ flx/flx mouse post‐Cre/GFP injection given alternating boluses of phenylephrine (PE) and sodium nitroprusside (SNP). (B) An example of a best‐fit HR and systolic BP baroreflex curve constructed from data points obtained. (C) Mean baroreflex curves (bold lines) for mice injected with Cre/GFP and GFP AVV (*n* = 5 in each group). Bold points represent mean HR 50 and SBP 50 for each group.

G*α*_O_ flx/flx mice were next studied under urethane anesthesia following injections of Cre/GFP AVV (*n* = 5) and GFP AVV (*n* = 5). In these conditions, G*α*_O_ flx/flx mice had significantly higher HR post‐Cre/GFP AVV injections compared to GFP AVV injections (629 ± 3 vs. 598 ± 5 bpm, *P* = 0.002, *t* test) with no difference in systolic BP (111 ± 2 vs. 115 ± 4 mmHg, *P* = 0.074). This was associated with an increase in baroreflex gain (0.65 ± 0.07 vs. 0.35 ± 0.01** **bpm mmHg^−1^, *P* = 0.012) (Fig. 7; Table 3).

**Table 3:** Summary data of parameters derived from baroreflex curve for mice with conditional deletion of G*α*_O_ in the RVLM compared to controls

	Cre (*n* = 5)	GFP control (*n* = 5)	*P* value
HR min (bpm)	**604 ± 2**	**575 ± 9** *	**0.028**
HR 50 (bpm)	**629 ± 3**	**598 ± 5** **	**0.002**
HR max (bpm)	**654 ± 6**	**620 ± 4** *	**0.017**
HR range (bpm)	50 ± 6	48 ± 7	0.451
SBP min (mmHg)	50 ± 4	43 ± 6	0.399
SBP 50 (mmHg)	115 ± 4	101 ± 2	0.074
SBP max (mmHg)	155 ± 5	153 ± 5	0.850
BP range (mmHg)	114 ± 6	107 ± 2	0.325
BR gain (bpm mmHg^−1^)	**0.65 ± 0.07**	**0.35 ± 0.01** *	**0.012**

Data are expressed as means ± SEM. Values highlighted in bold are significantly different when compared to controls. *P < 0.05, **P < 0.01. RVLM, rostral ventral lateral medulla; HR, heart rate; SBP, systolic blood pressure; BP, blood pressure.

### Ventricular excitability in conditions of Gα_O_ deletion in the RVLM

**Figure 8:**
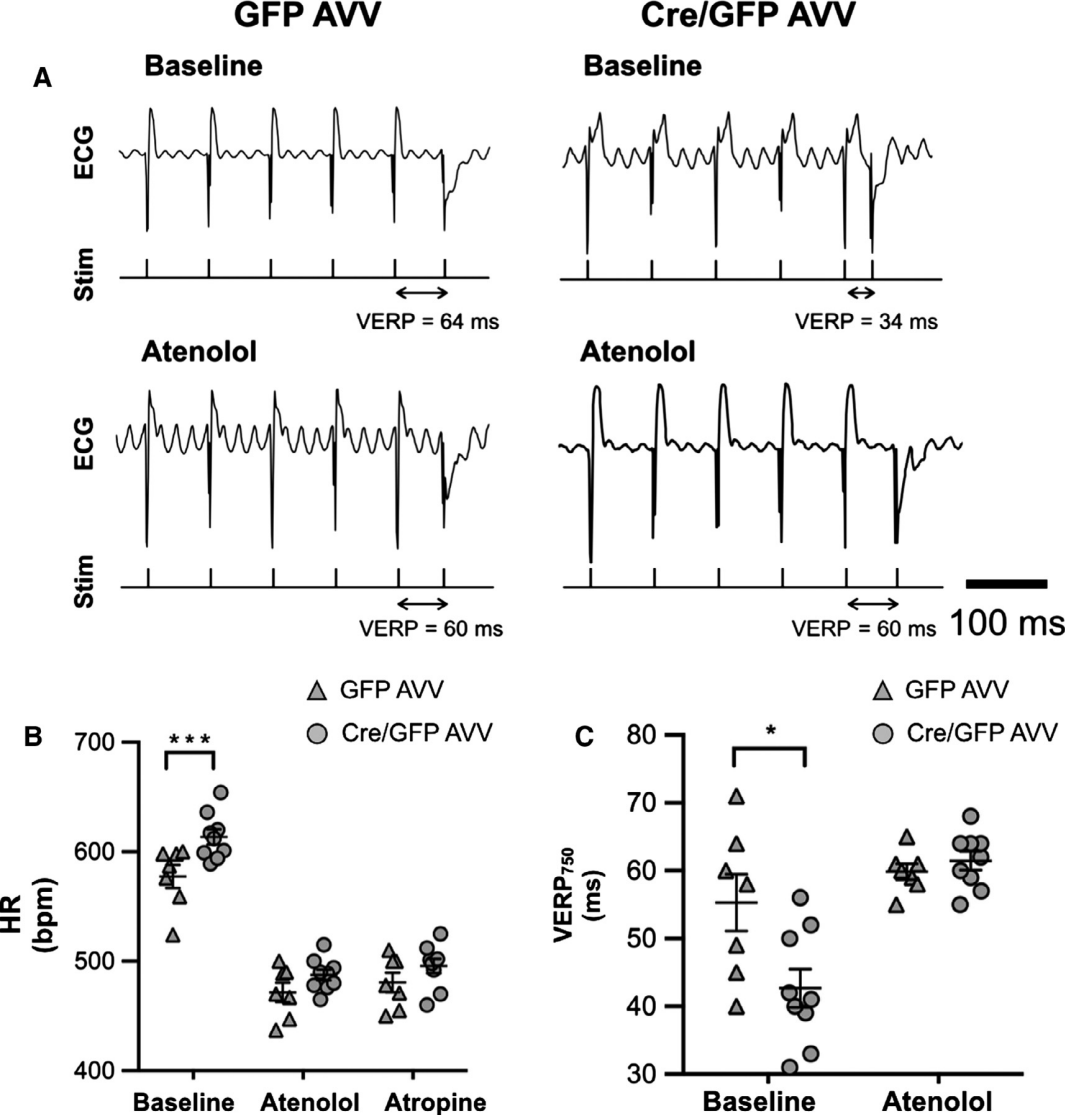
Effect of autonomic blockade on heart rate and ventricular excitability. (A) Representative in vivo cardiac electrophysiology data produced by programmed electrical stimulation showing an example of right ventricular effective refractory period (VERP) in G*α*_O_ flx/flx mouse injected with Cre/GFP adenoviral vectors (AVV) compared to control GFP AVV with lengthening of VERP on administration of atenolol. (B) Comparison of HR and (C) VERP_750_ between G*α*_O_ flx/flx mice post‐GFP (GFP,* n* = 7) and Cre/GFP (Cre, *n* = 9) AVV injections at baseline and after administration of atenolol 1 mg kg^−1^ ip ± atropine 1 mg kg^−1^ ip. Data expressed as mean ± SEM. *P <* *0.05, ***P <* *0.001. ECG, surface ECG; Intracardiac, intracardiac electrograms; Stim, pacing stimulus.

Cre/GFP AVV (*n* = 9)‐ and GFP AVV (*n* = 7)‐injected mice then underwent programmed electrical stimulation. The difference in resting HR was abolished following administration of atenolol with no effect on the HR with additional administration of atropine. Cre/GFP AVV‐injected mice had significantly reduced VERP_750_ compared to controls (VERP_750_ 43 ± 3 vs. 55 ± 4 ms, *P* = 0.033, Mann–Whitney *U*) and this difference in VERP_750_ was abolished by beta‐adrenoceptor blockade with atenolol (Fig. 8).

## Discussion

G*α*_O_ is the major inhibitory heterotrimeric G protein expressed in the brain and mediating inhibitory signaling by several neurotransmitters. In this study, we investigated whether G*α*_O_‐mediated signaling is important in the central nervous mechanisms controlling cardiovascular activities focusing on the RVLM presympathetic circuits. Our results show that mice with deletion of G*α*_O_ in the presympathetic circuits of the brainstem (RVLM) display normal HR and arterial BP profile under resting conditions. However, when stressed in a novel environment or studied under general (urethane) anesthesia, these mice show an elevated HR and abnormal hemodynamic responses during systemic hypoxia and hypercapnia as well as increased baroreflex sensitivity. Pharmacological autonomic blockade using atenolol and atropine suggested that this phenotype is largely due to an increase in sympathetic tone. Finally, this increase in sympathetic tone to the heart was found to be associated with alterations in ventricular electrophysiology as evidenced from reduced VERP.

### Deletion of Gα_O_ proteins in the RVLM does not affect diurnal HR or BP under resting conditions

There were no significant differences in HR and BP profile after G*α*_O_ deletion in the presympathetic area of the brainstem in conscious freely moving mice housed in standard conditions. This observation is in agreement with the findings obtained in rats where permanent experimental perturbations within the C1 are likely to be compensated as previously reported (Schreihofer et al. 2000; Madden and Sved 2003). These data suggest that impairment of RVLM function in controlling HR and BP can be fully compensated for in the awake and unstressed experimental animals.

### Altered hemodynamic responses to hypoxia and hypercapnia in mice with G*α*_O_ deletion in the RVLM

An increasing body of evidence is accumulating on the importance of the presympathetic CNS circuits, and more specifically the RVLM, in mediating body responses to a variety of physiological and pathological stressors (Guyenet et al. 2013). Our data add to this body of evidence of the key role played by the RVLM mechanisms under the conditions of environmental stress including hypoxia and hypercapnia. In addition, it would appear that inhibitory signaling in the brainstem mediated by G*α*_O_ proteins is an important mechanism restraining sympathetic activity. For example, the RVLM is activated by increased dorsomedial hypothalamic activity in response to psychological stressors (Nalivaiko and Blessing 2001; Madden and Morrison 2004). This increased sympathetic activity is reversibly inhibited by selective serotonin‐1A (5‐HT1A) receptor agonist 8‐OH‐DPAT (Nalivaiko et al. 2005; Horiuchi et al. 2008; Ngampramuan et al. 2008). 5‐HT1A receptors are one of the many GPCRs in the brainstem that signal via inhibitory G*α*_i/o_. In addition, GABA and enkephalins can tonically inhibit the RVLM potentially through GPCR pathways (Bowman et al. 2013). GABA, 5‐HT, and adenosine are inhibitory neurotransmitters that have also all been shown to be released in the brainstem during hypoxia (Richter et al. 1999). Loss of inhibitory signaling mediated by G*α*_O_ could potentially explain the augmented HR response during the hypoxic challenge. The corelease of GABA with glutamate with similar temporal kinetics (Richter et al. 1999) suggests a role for GABA as a likely mediator of the restraining influence to the excitatory effects of glutamate during hypoxia. Indeed, there is evidence that GABAergic inhibitory mechanisms are important in modulating RVLM activity during hypoxia in rat neonatal brainstem preparations (Boychuk et al. 2012).

### Increased sympathetic tone and its effect on ventricular excitability

Mice studied under urethane anesthesia have previously been shown to have reduced arterial pressure and increased HR compared to the conscious state (Desai et al. 1997; Paton and Butcher 1998). Similar findings were obtained here with mice being hypotensive and generally more tachycardic. In general, anesthesia results in a reduction of vagal tone, but urethane seems to lead to relative sparing compared to other anesthetics (Machhada et al. 2015). However, the RVLM does contain vagal preganglionic motoneurons (Izzo et al. 1993). Sequential challenges with atenolol and atropine revealed low vagal tone and demonstrated that sympathetic activity indeed dominates HR control in mice under urethane anesthesia. This suggests that increased sympathetic outflow (rather than vagal withdrawal) is responsible for the increased HR and enhanced baroreflex sensitivity seen in mice with deletion of G*α*_O_ in the RVLM.

It is well known that the autonomic nervous system modulates cardiac excitability. Increase in sympathetic tone to the heart has been shown to reduce action potential duration with a corresponding increase in conduction velocity, resulting in a change in the restitution properties of action potential duration and dispersion of ventricular repolarization and increased susceptibility to ventricular arrhythmia (Ng et al. 2009; Finlay et al. 2016). Our data add to this body of evidence and demonstrates how alterations in central nervous mechanisms controlling cardiorespiratory activity can alter ventricular excitability.

### G protein specificity in vivo

Parker et al. (2012) has previously shown that G*α*_i2_ and G*α*_O_ are the most ubiquitous inhibitory G proteins in the RVLM. In keeping with this, our data suggest an important functional role for G*α*_O_. We cannot exclude a role for G*α*_i2_ as this was not studied directly with a conditional KO of G*α*_i2_ in the brainstem. Our own previous studies showed an effect on cardiac function in global G*α*_i2_ KO mice, though we have ascribed this largely to direct effects on cardiac tissue (Zuberi et al. 2008, 2010). Other investigators have shown a potential role for G*α*_i2_ in the rat in mediating hypertensive responses (Wainford et al. 2015; Carmichael et al. 2016).

### Limitations

The A1 and A5 cell groups and the vagal preganglionic neurons lie in close proximity to the RVLM in the ventral medullary brainstem region (Strack et al. 1989). Although great care was taken to target viral delivery to the RVLM area, given the nonspecific nature of the CMV promoter, it is likely that some of the neurons in the neighboring regions would have undergone Cre‐mediated deletion of G*α*_O_. However, given that the subsequent histological analysis demonstrated expression of GFP to be distributed among TH‐immunoreactive neurons in the RVLM region, the observed phenotype is likely to be mainly due to the loss of G*α*_O_‐mediated signaling within the RVLM presympathetic circuits.

The relative contributions of the sympathetic and parasympathetic limbs of the autonomic nervous system to the phenotype observed were assessed by pharmacological blockade. These are indirect measures of autonomic function. A more direct approach would involve direct recordings of efferent nerve activity such as of the renal sympathetic nerve (Ma et al. 2002) or other experimental paradigms such as in the working heart brainstem preparations (Paton 1996).

The precise mechanism by which loss of G*α*_O_ at the RVLM results in alterations in autonomic tone remains unknown. We suggest that G*α*_O_ is important in mediating inhibitory influences on the RVLM with loss of this resulting in disinhibition and increased sympathetic outflow. G*α*_O_‐coupled receptors can cause pre‐ or postsynaptic inhibition either by inhibiting calcium channels or activating G‐protein‐gated K^+^ channels, respectively (Dolphin 1998; Luscher and Slesinger 2010). The specific neurotransmitter(s) and exact mechanism is a topic for further work in the follow‐up studies.

## Conclusion

In summary, the data obtained in this study suggest that G*α*_O_‐mediated signaling within the presympathetic circuits of the RVLM contributes to the autonomic control of the heart. G*α*_O_ deficiency in the RVLM is associated with abnormal cardiovascular responses to stress, altered cardiovascular reflexes, and ventricular excitability.

## Conflicts of Interest

The authors have no conflicts of interest to declare.
